# Eco-Friendly Sustainable Synthesis of Graphene Quantum Dots from Biowaste as a Highly Selective Sensor

**DOI:** 10.3390/nano12203696

**Published:** 2022-10-21

**Authors:** Aumber Abbas, Qijie Liang, Saleem Abbas, Maryam Liaqat, Shabnum Rubab, Tanveer A. Tabish

**Affiliations:** 1 School of Engineering, Newcastle University, Newcastle upon Tyne NE1 7RU, UK; 2 Songshan Lake Materials Laboratory, University Innovation Park, Dongguan 523808, China; 3 Department of Physics, University of Okara, Okara 56300, Pakistan; 4 Department of Chemistry, University of Sargodha, Ex. Mianwali Campus, Mianwali 42200, Pakistan; 5 Radcliffe Department of Medicine, University of Oxford, Oxford OX3 7BN, UK; 6 Department of Engineering Science, University of Oxford, Parks Road, Oxford OX1 3PJ, UK

**Keywords:** graphene quantum dots, biowaste, sustainable synthesis, fluorescence sensors

## Abstract

Graphene quantum dots (GQDs) have generated a great deal of scientific interest due to their bright fluorescence, good biocompatibility, minimal toxicity and fascinating physicochemical features. However, the ultimate issues regarding the acidic contaminations and high synthesis cost of GQDs remain open challenges for their real-world applications. Herein, we report an eco-friendly, acid-free and sustainable method for the preparation of GQDs using a cost-efficient, and renewable carbon source, ‘biomass-waste’, which simultaneously solves the risk of contamination from strong acids and high expenditure initiated by expensive precursors. The results demonstrate that GQDs possess a size range of 1–5 nm with an average size of ~3 ± 0.4 nm and a thickness of ~1 nm consisting of 1–3 layers of graphene. As-prepared GQDs demonstrate fascinating size-dependent optical properties and considerable surface grafting. Due to their intriguing optical properties, these GQDs are employed as fluorescence probes to detect ferric ions. A focused and sensitive sensor is developed with a detection limit down to 0.29 µM. This study emphasizes the need for using a reasonably green process and an inexpensive biomass precursor to create high-value GQDs that hold great potential for use in photocatalytic, bioimaging and real-world sensing applications.

## 1. Introduction

Graphene quantum dots (GQDs) have attracted extensive research interest due to their intriguing properties and promising applications. GQDs are single- or few-layer graphene derivatives with lateral size less than 100 nm. Due to their zero dimensionality, quantum confinement and edge effects, GQDs exhibit extraordinary properties such as large surface area, excellent water solubility, tunable bandgap, controllable photoluminescence, high biocompatibility, low cytotoxicity, and excellent photostability. GQDs are extremely desirable in the fields of materials, physics, chemistry, biology, and other multidisciplinary avenues because of the above-mentioned exceptional features. Typically, GQDs’ distinct optical feature makes them suitable building blocks for wide-ranging photo catalysis applications. [1,2,3], photo electrical conversion [4,5], bio imaging [6,7,8], optoelectronic devices [5,9,10] and light-emitting diodes (LEDs) [11,12]. Therefore, it is extremely desirable to build high-quality, affordable, and sustainable GQDs on a broad scale.

Several reports have been presented on the synthesis of GQDs; however, the cost-effective, green and sustainable synthesis of GQDs remains scarce in the scientific literature. The traditional methods used for the synthesis of GQDs include acid oxidation [13,14], hydrothermal treatment [15], microwave treatment [16], electrochemical approach [17], as well as pyrolysis [18] and carbonization [19] with different carbon sources. These carbon sources include graphene, graphene oxide, graphite, carbon nanotubes, carbon fiber, citric acid, glucose and coal. Generally, most of the methods employed to produce GQDs involve strong acids, expensive starting materials and/or are limited to only laboratory scale productions [20,21]. For example, Wang et al. reported the so-called large-scale synthesis of GQDs from rice husk biomass [22], while the actual precursor material used was just 50 mg. In addition, this approach involves lengthy treatment with strong acids. Suryawanshi et al. also presented the large-scale synthesis of GQDs from biomass; however, the approach was limited to high temperature treatment in strong acids. Ye et al. reported coal to be an abundant source of GQDs but the treatment with strong acids was still inevitable [23]. Shin et al. reported the mass production of GQDs from graphite under strong acidic conditions [24]. The treatment with strong acid makes the whole process quite complex and lengthy. To ensure acid removal, a lengthy purification process is required, which involves neutralization with a strong base and produces a large amount of salt. The salt removal takes several days of dialysis. Therefore, it is strongly recommended that an acid-free technique be developed. An acid-free method for making GQDs was devised by Shin et al.; however it was only applicable to commercially synthesized precursors such as carbon nanotubes and carbon film, and the amount of finished product was extremely minimal [25]. Despite these efforts, the final product is still very expensive for practical application. Therefore, the ultimate issues regarding the acidic contaminations and high synthesis cost of GQDs remain open challenges for their real-world applications. To this end, we present an acid-free approach for scalable production of GQDs from inexpensive precursor for their wide-spread practical applications. The main difference between the reported studies and our work is the use of green, abundant, renewable, sustainable and low-cost precursors for GQDs production in combination with an acid-free scalable approach.

It is very desirable to create and develop sensors that can detect heavy metal ions in biological and environmental systems. Due to the intriguing optical characteristics of GQDs, a brand new class of fluorescence sensors has been created using GQDs [26,27,28]. Due to iron’s crucial role in environmental systems and human survival, several researchers have shown a lot of interest in detecting and removing ferric ions (Fe^3+^) [29,30]. Since iron accretion was discovered in Parkinson’s disease (PD) patients’ neurons, it is imperative to monitor the Fe^3+^ concentration in these patients [31]. Similarly, Fe^3+^, as one of the main water contaminants, contributes to environmental contamination [32]. In order to tackle these challenges, iron concentrations in biological and environmental systems must be precisely recorded, documented, and monitored [33]. The photoluminescence quenching phenomena of GQDs by Fe^3+^ can be used to accomplish these aims [29,34]. GQDs are an effective instrument for tracking toxic metal ions in the environment due to their straightforward and sensitive fluorescence-based sensing capabilities. Despite several reports on GQD-based Fe^3+^ sensing, these sensors are far from realizing real-world applications. These sensing platforms still require further investigation in various aspects; for example, cost is one of the most important factors to consider. In this study, we address this issue by employing a cost-effective precursor while controlling the product quality.

Here, we offer an acid-free eco-friendly procedure for the production of GQDs from biomass waste using potassium monopersulphate (Oxone) as an oxidant. By utilizing the Oxone oxidant, it is possible to generate a controlled solvothermal redox process that combines radical oxidation and solvothermal reduction. Thus, the hydroxyl (OH^•^) or sulphate (SO4^•−^) radicals can be generated under control. The main mechanism for the scission of carbon–carbon bonds consists of these radicals in conjunction with solvothermal reaction. During this process, the carbon domains in the biomass waste can be repeatedly broken down. The carbon domains finally convert into small-sized GQDs under the repeated cycles of oxidation, cleavage and reduction. These GQDs have powerful optical characteristics that make them ideal for applications such as sensing. These GQDs are used as a fluorescence probe to detect Fe^3+^ as a proof of concept.

## 2. Experimental Methods

### 2.1. Materials

Used black tea bags (PG Tips) were gathered from a local shop. Potassium monopersulphate (Oxone, 2KHSO_5_·KHSO_4_·K_2_SO_4_), sodium hydroxide (NaOH) and N, N-Dimethylformamide (DMF, (CH_3_)_2_ NC (O)H) and were purchased from Fisher Scientific, Loughborough, United Kingdom, and used without any further modification. Millipore, based in the United Kingdom, provided the polyvinylidene fluoride (PVDF) micro porous filtering membranes (0.1 µm and 0.025 µm). GQDs were purified using 1 kD MWCO dialysis bags (Spectrum Labs, Breda, The Netherlands; item number 132640). For the morphological analysis, mica discs (AGF7013, Agar Scientific, Essex, United Kingdom) and TEM grids with ultra-thin carbon film (CF-400-Cu-UL, Electron Microscopy Sciences, Hatfield, PA, USA) were employed. Water that has been de-ionized (DI) was used to create all the solutions.

### 2.2. Preparation of GQDs

**Figure 1:**
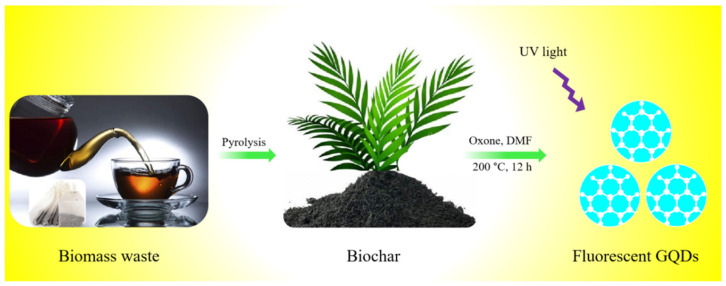
Schematic illustration of the development of GQDs from biomass waste. Biomass waste is first converted into biochar via pyrolysis and then treated at 200–250 °C for several hours in the presence of Oxone and DMF to produce GQDs.

Blue fluorescent GQDs were synthesized from spent tea leaves as a green biomass waste. Figure 1 shows the schematic illustration of synthesis process for the preparation of GQDs from biomass waste.

The spent tea leaves were acquired from used tea bags and properly washed in deionized (DI) water to eliminate the organic material (such as color) and dust particles before being allowed to air dry for one day. To release the retained moisture, the samples were subsequently oven-dried at 80 °C for an additional 12 h. The dried materials were ground to fine powder (<90 µm) using a commercial grinder. The powder sample was placed in a stainless container and subjected to pyrolysis at 500 °C for 3 h followed by cooling down to room temperature naturally. Subsequently, the biochar samples were collected and washed to remove any impurities. Washing was performed by boiling the biochar in 1M HCl, followed by hot DI water washing, and finally, normal DI water washing. The product yield of biochar was calculated to be ~35%. Approximately 1 g of the biochar powder, 2 g of Oxone, and 100 mL of DMF were mixed, and the mixture was then sonicated for an hour. Subsequently, the mixture was placed in an autoclave coated with Teflon and cooked for variable lengths of time at varying temperatures (200 and 250 °C for 8 h and 12 h). Afterwards, the reactor was allowed to gradually cool to ambient temperature without any more heating. The resulting brown solution was filtered across a 0.1 µm PVDF microporous membrane and dialyzed in a dialysis bag for a whole day to remove DMF solvent and any impurities. Consequently, GQDs were collected from dialysis bags showing a pale-yellow color. Although not definitive, the pale-yellow color of the solution in visible light is an indication of the GQD formation [16,35].

### 2.3. Characterization

The samples’ structure, morphology, and characteristics were studied utilizing various analytical methods. Transmission electron microscopy (TEM and FEI, operated at 300 kV) and high-resolution transmission electron microscopy (HRTEM) were used to examine the morphology, microstructure, and size of the samples. The diameter and size distribution of GQDs were determined from TEM images using the “ImageJ Program, version 1.46r, developed by Wayne Rasband at the National Institute of Mental Health, Bethesda, MD, USA”. The thickness of the GQDs was measured using atomic force microscopy (AFM, XE-15, Park Systems, Nottingham, United Kingdom), and the number of graphene layers was counted from AFM images using the ‘Gwyddion 2.55′ program. The structure and electronic properties of samples were studied using Fourier-transform infrared (FTIR) spectroscopies. The FTIR spectra were recorded using a Bio-Rad FTIR spectrometer with a Diamond ATR.KBr tip (Cary 630 FTIR, Agilent Technologies Inc., Santa Clara, CA, USA). The pH of the samples was checked using a pH meter (Mettler Toledo, Five Easy pH meter). The absorption and fluorescence spectra were recorded using the Shimadzu RF-6000 Spectrofluorophotometer and Shimadzu UV-1800 spectrophotometer, (SHIMADZU, Kyoto, Japan) respectively.

## 3. Results and Discussions

### 3.1. Optical Property Study

**Figure 2:**
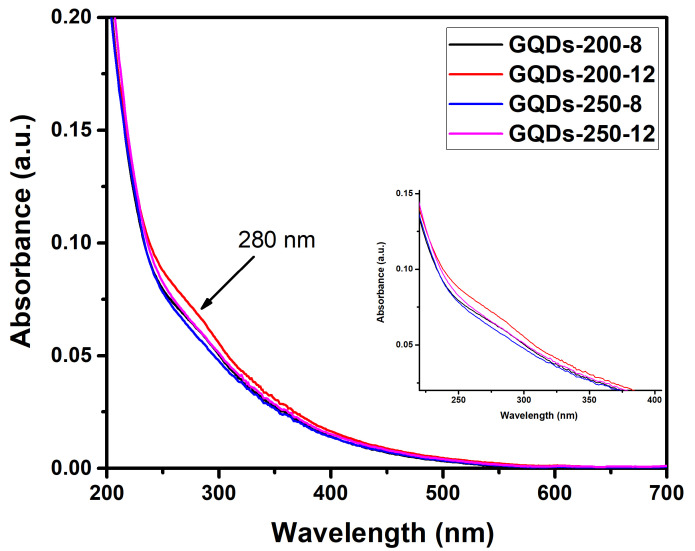
Ultraviolet–visible (UV–Vis) spectra of GQDs-200-8, GQDs-200-12, GQDs-250-8 and GQDs-250-12 showing a noticeable absorption peak at about 280 nm.

Ultraviolet–visible (UV–Vis) and photoluminescence spectroscopies were used to investigate the optical characteristics of GQDs. Figure 2 illustrates the UV–Vis absorption spectra of GQDs, which exhibits a significant UV absorption with a peak around 280 nm for a series of GQDs (inset in Figure 2). The strong absorption band below 300 nm is attributed to the π–π* transition in aromatic carbon domains. The comparison of absorption peaks of different GQDs shows a strong absorption for the GQDs prepared over a reaction time of 12 h (namely, GQDs-200-12 and GQDS-250-12). The strong absorption of GQDs-200-12 indicates their superior optical properties. The *n*–π* transition is thought to be responsible for the bands over 300 nm which is commonly observed in graphene systems. The non-bonding or “*n*” electrons are the unpaired electrons on the oxygen atom of the carbonyl group (C=O). When one of the unpaired oxygen electrons is propelled into the antibonding π* orbital, it transitions from *n* to π*. These results suggest that the aromatic carbon domains and surface oxygen states are responsible for the UV absorbance in these GQDs. The Tauc plots of the absorbance spectra [36] were used to calculate the band gaps of GQDs-200-12 and GQDs-250-12, which were found to be 5.43 eV and 5.5 eV, respectively. The concept of band gap is useful in understanding the interaction of light with matter. The band gap is a useful predictor of wavelength of light that will be absorbed by the material. This relationship is inverse, meaning that a light of shorter wavelength will be absorbed by the GQDs with larger band gap.

**Figure 3:**
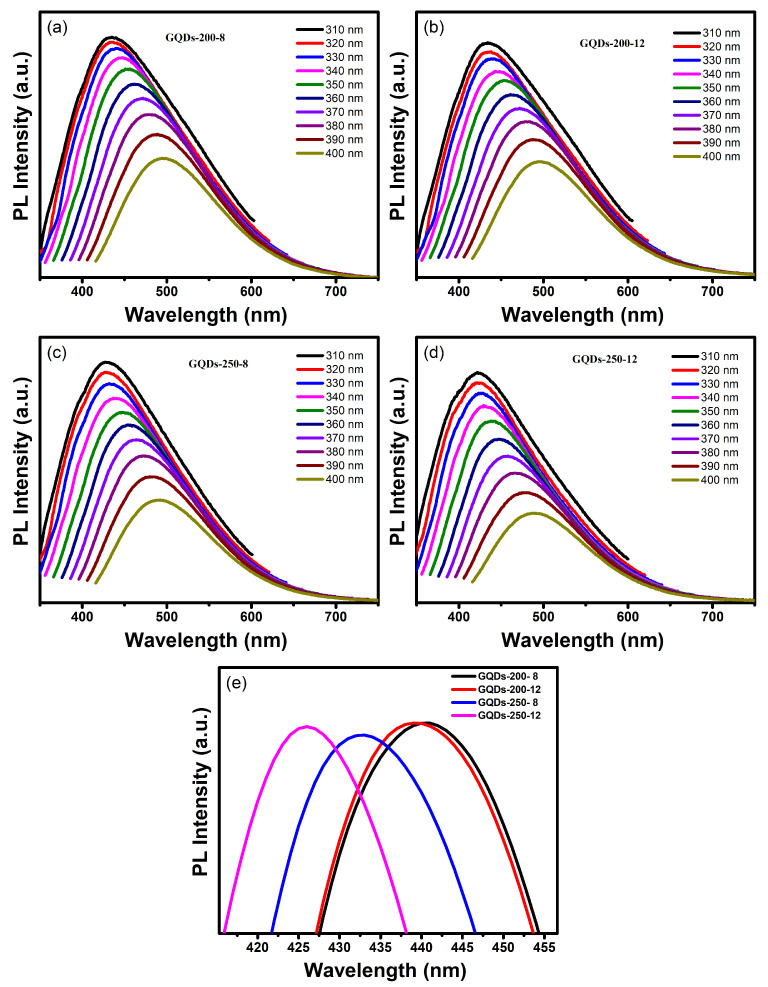
Photoluminescence emission spectra at a range of excitations from 310–400 nm for (**a**) GQDs-200-8, (**b**) GQDs-200-12, (**c**) GQDs-250-8 and (**d**) GQDs-250-12. (**e**) The comparison of emission peak position for various GQDs at same excitation of 330 nm.

The optical properties of these GQDs were further examined by studying their photoluminescence emission at a wide range of excitations. When stimulated by a 310 nm laser light, the GQDs display excitation at a wavelength of roughly 400 nm (Figure 3). When the emission wavelength is longer than the excitation wavelength, Stokes shift occurs. The GQDs’ photoluminescence intensity decreases with increasing the excitation wavelength and shows a red shift. As a result of distinct functional groups creating various emissive states, the GQDs produced in this study display excitation dependent photoluminescence emission [16,26,34]. A variation in excitation wavelength causes a shift in emission maxima. When the excitation wavelength is raised from 300 to 400 nm, the emission peak site moves and exhibits a red shift of around 50 nm. Differing surface functional groups or GQDs with different sizes are believed to be the cause of this red shift [22].

The comparison of the photoluminescence spectra of different types of GQDs prepared at 200 °C and 250 °C demonstrates that the emission peak shows a blue shift with increase in the reaction time and temperature (Figure 3c,d). This is further verified in Figure 3e. It can be observed in Figure 3e that the strongest emission peak shifts from 440 nm in GQDs-200-8 to 425 nm in GQDs-250-12 due to increase in reaction time and temperature. This shifting of emission peak toward higher energy region indicates the increase in their band gap. The optical band gap can be calculated from the Tauc plots of absorption spectra. The band gaps calculated from absorption spectra of GQDs-200-8, GQDs-200-12, GQDs-250-8 and GQDs-250-12 are 5.42, 5.43, 5.45 and 5.5, respectively. It is well known that the band gap increases with a decrease in the size of GQDs, leading to a blueshift in their PL emission [37,38,39]. These results suggest that the GQDs prepared after 12 h of hydrothermal reaction at 250 °C have smaller sizes and exhibit larger band gaps. The small size and large band gap lead to improved optical properties of these GQDs.

**Figure 4:**
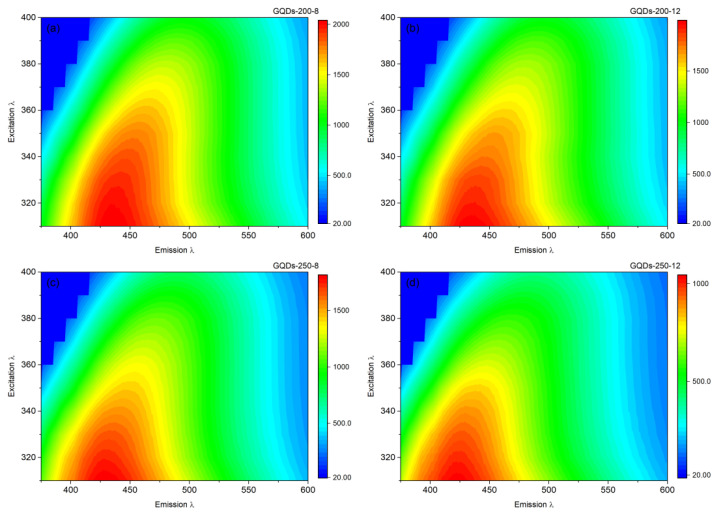
Contour maps of (**a**) GQDs-200-8, (**b**) GQDs-200-12, (**c**) GQDs-250-8 and (**d**) GQDs-250-12 for photoluminescence emission at a range of excitations from 310 to 400 nm.

Further investigations on the PL spectra of these GQDs were conducted with the help of contour maps presented in Figure 4. The contour maps illustrate that the strong emission indicated by red region spans over a wide range for GQDs-200-8, while this emission confines to a small region with an increase in the hydrothermal duration to 12 h for GQDs-200-12. The emission range of GQDs-250-8 and GQDs-250-12 is further confined to a very short range. These results can be confirmed from the size of the red regions of contour maps associated with the highest emission range.

The red region of GQDs-200-8 and GQDs-200-12 are relatively large and expand over a wide range, while the red regions of GQDs-250-8 and GQDs-250-12 are very small. These results indicate the size distribution of these GQDs leading to a wide or narrow emission range. The wide emission range can be attributed to the large size distribution, while the confined emission can be attributed to a very narrow size distribution [39,40]. Thus, we propose that the GQDs-250-12 exhibiting the most strong and narrow emissions consist of most narrow size distribution among various types of GQDs prepared. These findings are rational since longer hydrothermal duration and high temperature would lead to the generation of small GQDs. The product yield of the GQDs-250-12 based on biochar was determined to be ~26% and yield based on biomass precursor was calculated to be ~9.12%. Due to their promising optical properties, GQDs-250-12 were mainly focused on for further characterization, investigation and application.

### 3.2. Morphology Study

**Figure 5:**
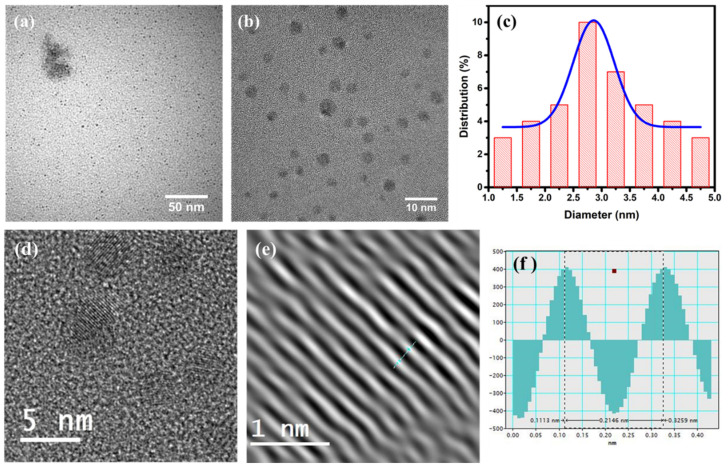
Transmission electron microscopy images of GQDs-250-12 (**a**) at low magnification, (**b**) at high magnification and (**c**) particle size distribution of GQDs. (**d**) HRTEM image of GQDs, (**e**) magnified image of lattice fringes and (**f**) line profile showing the distance between adjacent lattice fringes.

The morphology and nanostructure of the GQDs-250-12 prepared by solvothermal carbonization of spent tea were characterized by AFM and TEM microscopy. Figure 5 depicts the TEM image of GQDs with a uniform shape and size distribution. It is possible to see the quantum dots’ precise size and uniform dispersion without any aggregation. The high-resolution image in Figure 5b further confirms their spherical shape. The careful size measurements of as-synthesized GQDs are presented in Figure 5c and exhibit a size distribution of ~3 ± 0.4 nm. The HRTEM picture (Figure 5d,e) demonstrates remarkable crystallinity of the as-prepared GQDs, with a lattice parameter of ~0.21 nm (Figure 5f), which corresponds to the distance between the hexagonal lattice planes of carbon (1100).

**Figure 6:**
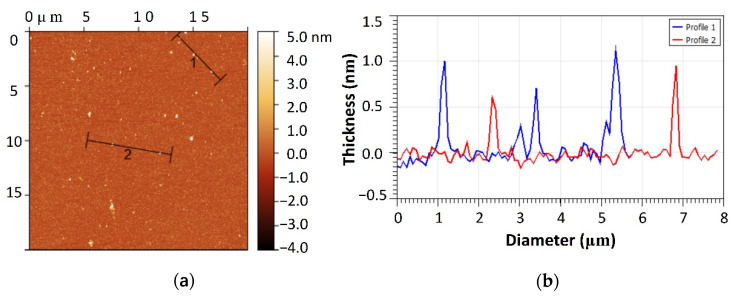
(**a**) AFM image of GQDs-250-12 and (**b**) corresponding height profiles of the lines marked in (**a**).

The successful preparation of GQDs was further verified by AFM in order to learn more about their thickness. The AFM image of GQD-250-12 is presented in Figure 6, which reveals the topographic morphology of GQDs. According to the analogous height profile, the thickness of as-prepared GQDs is 0.6 to 1 nm corresponding to 2–3 layers of graphene. These findings suggest that as-prepared GQDs are very thin discs of graphene consisting of a few layers. These results further verify the graphene nature of these quantum dots in contrast with their carbon quantum dots counterpart which are spherical particles of carbon with plenty of carbon layers.

### 3.3. Structural Examination

**Figure 7:**
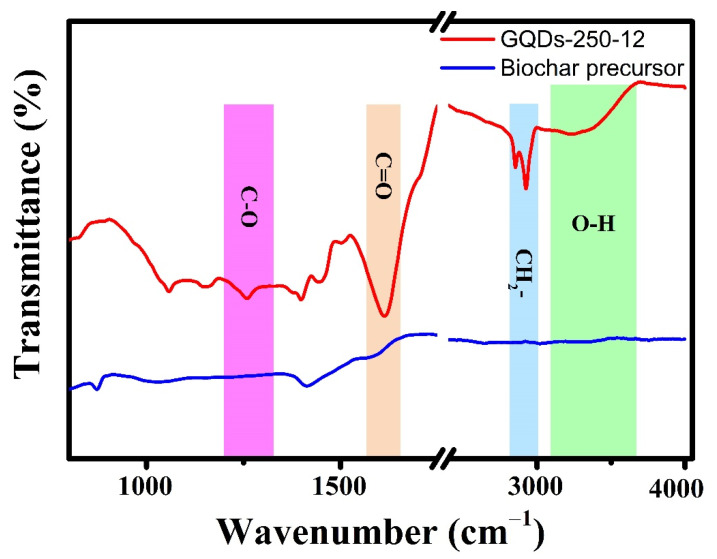
FTIR spectra of GQDs-250-12 and biochar precursor indicating the growth of functional groups on the surface of GQDs during synthesis.

Using FTIR spectroscopy, the structural and electrical characteristics of as-synthesized GQDs-250-12 were studied. The FTIR of a typical sample (Figure 7) reveals a high absorption of oxygenated functional groups on the surface of GQDs [41]. The spectra demonstrate that the GQDs contain abundant surface functional groups, such as hydroxyl (O-H @ 3300 cm^−1^), carbonyl (C=O @ 1616 cm^−1^), and epoxy (C-O @ 1250 cm^−1^) groups [41,42]. The GQDs have multiple hydroxyl, carboxyl and other oxygenated groups on their surface, which provide them hydrophilic characteristics and boost luminescence. Due to the presence of abundant oxygen-bearing functional groups, GQDs make stable suspensions and are readily dispersible in water.

Due to their extensive surface functionalization and strong optical properties, these GQDs can be applied to develop a selective and sensitive sensor for biological systems and environmental monitoring.

### 3.4. Application as a PL Sensor

As mentioned above, as-prepared GQDs-250-12 have many surface functional groups and high fluorescence, which gives them a hydrophilic nature and makes them soluble in water. Due to their good solubility, strong optical properties, and superior surface grafting, GQDs can be employed as a novel type of optical sensing probe [26,27,28,43].

**Figure 8:**
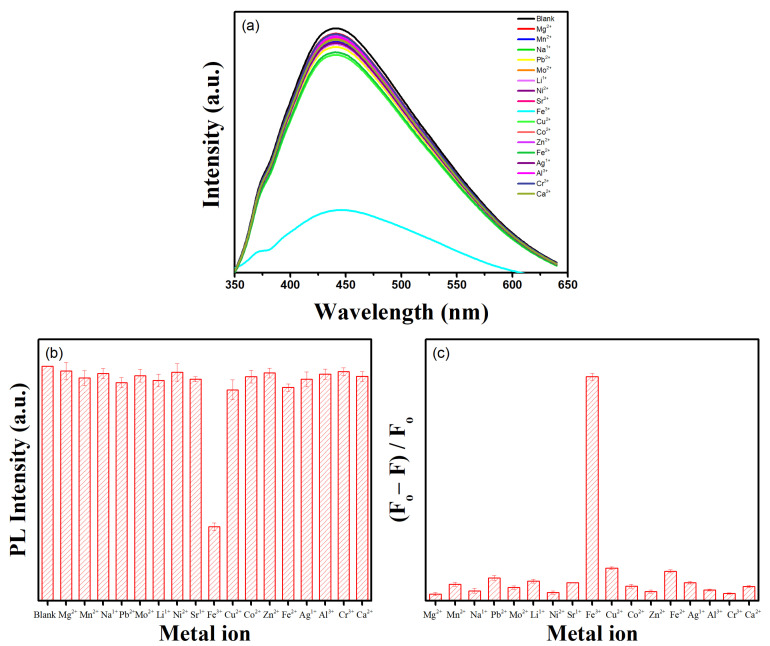
(**a**) Fluorescence spectra of GQDs-250-12 solution in the presence of different metal ions at 340 nm excitation. (**b**) Comparison of the emission intensity of GQDs in the absence and presence of various metal ions. (**c**) Comparison of the affinity of a range of metal ions towards GQDs.

By analyzing the quenching behavior of freshly synthesized GQDs-250-12, the sensitive and selective sensing of Fe^3+^ was investigated in the present study. With the addition of numerous metal ions, including Co^2+,^ Ca^2+^, Ag^1+^, Al^3+^, Cr^3+^, Fe^2+^, Cu^2+^, Zn^2+^, Fe^3+^, Sr^2+^, Pb^2+^, Ni^2+^, Mo^2+^, Li^1+^, Na^1+^, Mn^2+^, and Mg^2+^, this excitation value was selected to investigate the photoluminescence quenching behavior of the GQD solution (10 mg mL⁻^1^). A 100 µM solution of different metal ions was added to GQDs solution with incubation for 2 min, and the PL emission spectrum was recorded at 340 nm excitation. As seen in Figure 8a, most of the metal ions kept the PL emission spectra of the GQDs solution steady, except for Pb^2+^, Cu^2+^, and Fe^2+^, where a minor decrease was seen. Interestingly, when Fe^3+^ ions were added, the PL emission of GQDs was substantially quenched, indicating the strong quenching tendency of Fe^3+^. To examine the quenching propensity of different metal ions, the intensity ratio (F/F_0_, where F_0_ and F are the photoluminescence intensities of GQDs in the absence and presence of separate metal ions, respectively) of GQDs solution was computed. Figure 8b shows that, compared with the control sample (blank, GQDs solution without any metal ions), only Fe^3+^ considerably reduces the intensity ratio of GQDs. These findings demonstrate the GQDs’ high selectivity for identifying Fe^3+^.

It is critical to comprehend the quenching mechanism in order to have further understanding of the strong quenching behavior of Fe^3+^. The surface functional groups for which Fe^3+^ has a strong affinity are hydroxyl/carboxyl groups present on the surface of GQDs. A stable molecule is created as a result of the attraction of hydroxyl/carboxyl groups on Fe^3+^ [32]. Fluorescence quenching occurs when an excited electron partially travels towards the d orbital of Fe^3+^ instead of relaxing radiatively [28,32]. Under identical experimental settings, the scientific affinity of several metal ions for GQDs was investigated. The findings (Figure 8c) show that among the seventeen different metal ions, Fe^3+^ has the highest affinity for GQDs. These findings suggest that these GQDs have a promising future in the field of selective Fe^3+^ practical detection.

**Figure 9:**
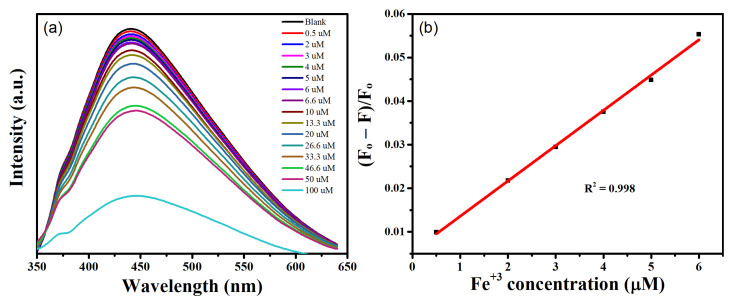
(**a**) Fluorescence spectra of GQDs (10 mg mL^−1^) with Fe^3+^ concentrations ranging from 0 to 100 µM, with an excitation wavelength of 340 nm and (**b**) Linear regression plot of fluorescence intensity and Fe^3+^ concentration in low concentration range of 0.5–6 µM.

In real-world sensing applications, sensitivity is a crucial component in addition to selectivity. The impact of various Fe^3+^ concentrations on the PL intensity of GQDs was carefully investigated in order to explore the sensitivity of the GQD-based sensor. Figure 9a shows how the PL intensity of the GQDs solution changed as varied amounts of Fe^3+^ (0.1 to 100 µM) were added. With a gradual increase in the quantity of Fe^3+^, a consistent drop in PL intensity is clearly seen. With a rise in Fe^3+^ concentration, PL quenching increases. These findings show that Fe^3+^ has considerable quenching efficiency and that GQDs are sensitive for Fe^3+^ detection.

Figure 9b shows the calibration plot for the quenching efficiency of Fe^3+^ measured by using the relation (F₀ − F)/F₀. The calibration plot reveals a sharp increase in quenching efficiency with rise in the concentration of Fe^3+^. Meanwhile, the quenching at lower range (0 to 6 µM) was further explored. As shown in Figure 9b, a strong linear relationship between quenching efficiency and Fe^3+^ concentration is found. The attainment of a superb fit and precise Fe^3+^ detection even at very low concentrations is shown by the linear regression result (R^2^) of 0.998. These results suggest that these GQDs can be used as an extremely sensitive and focused probe for Fe^3+^ detection.

The relation $\mathrm{LOD}=3\frac{\sigma }{S}$, where σ is the standard deviation of intercept and s is the slop of linear calibration plot, was used to establish the limit of detection (LOD). The LOD for this investigation was measured to be 0.29 ± 0.4 µM. The LOD for Fe^3+^ provided in this work is lower than that of numerous prior investigations, as seen in Table 1. The LOD obtained in this study is also considerably lower than the World Health Organization’s (5.36 µM) recommended limit for Fe^3+^ in drinking water [44]. Moreover, the iron levels recorded in a range of PD patients were 565–820 µg L^−1^, with an average of 675 µg L^−1^ [45]. The detection limit of our sensor ‘0.29 µM’ is equivalent to ~16.2 µgL^−1^, which is much lower than the lower limit of iron accumulation in PD patients. Therefore, we believe that our sensor is also suitable for monitoring trace levels of iron in PD patients.

These GQDs exhibit very low LOD for Fe^3+^ detection due to their strong fluorescence, superior purity, good water solubility, and precise surface grafting. These results point to the great sensitivity of the current GQD-based sensor for detecting Fe^3+^ traces in biological and environmental systems. We believe that the current GQDs can reliably detect Fe^3+^ in real-world sensing applications.

## 4. Conclusions

An acid-free, cost effective, relatively green, and sustainable synthesis of GQDs from biomass waste has been successfully accomplished. The present approach eliminates the risk of contamination from strong acids and high cost due to commonly used expensive precursors. According to the findings, GQDs have been produced with an average size of ~3 ± 0.4 nm and a thickness of 0.5–1 nm. As-prepared GQDs exhibit abundant surface functionalization and intriguing optical properties. These GQDs were used as a fluorescent probe for Fe^3+^ detection because of their intriguing optical characteristics. To selectively detect Fe^3+^, a sensor with a high sensitivity down to 0.29 ± 0.4 µM was developed. This straightforward, yet effective, method demonstrates the benefits of high purity, cost efficiency, sustainability, and the potential for scaling-up for generating high grade GQDs on an industrial scale. The current study emphasizes the significance of employing low-value biomass resources to produce high-value nanomaterials for practical sensing applications.

## Figures and Tables

**Table 1:** A comparison of various reported studies with the present work for the limit of detection of Fe^3+^ by GQDs.

Sensing Material	Synthetic Approach	Detection Range (µM)	LOD (µM)	Ref.
GQDs	Electrochemical exfoliation	0–80	7.22	[34]
PEG-GQDs	Hydrothermal	0–60	5.77	[46]
GQDs	Microwave treatment	0–50	2.5	[28]
GQDs	Chemical oxidation	0–60	0.45	[32]
CQDs	Thermal reaction	0–20	0.041	[47]
N-CQDs	Hydrothermal	0–250	0.75	[48]
GQDs	Hydrothermal	0–100	0.29	Present work

## Data Availability

All data included in this study are available upon request by contacting the corresponding author.
